# State-Space Formulation for Buckling and Free Vibration of Axially Functionally Graded Graphene Reinforced Nanocomposite Microbeam under Axially Varying Loads

**DOI:** 10.3390/ma17061296

**Published:** 2024-03-11

**Authors:** Dongying Liu, Junxiang Su, Li Zhao, Xudong Shen

**Affiliations:** 1 School of Civil Engineering, Guangzhou University, Guangzhou 510006, China; 2 School of Civil Engineering and Architecture, Zhejiang University of Science & Technology, Hangzhou 310023, China; 3 Huanjiang Laboratory, No. 7 Wenzhong Road, Taozhu Street, Zhuji 311816, China

**Keywords:** axially functionally graded GPL-reinforced microbeam, axially varying applied load, buckling, vibration, modified couple stress theory

## Abstract

This paper focuses on the size-dependent free vibration and buckling behaviors of the axially functionally graded (AFG) graphene platelets (GPLs) reinforced nanocomposite microbeams subjected to axially varying loads (AVLs). With various axial grading patterns, the GPL nano-reinforcements are distributed throughout the polymer matrix against microbeam length, and the improved Halpin–Tsai micromechanics model and the rule of mixture are adopted to evaluate the effective material properties. Eigenvalue equations of the microbeams governing the static stability and vibration are derived based on the modified couple stress Euler–Bernoulli beam theory via the state-space method, and are analytically solved with the discrete equilong segment model. The effects of axial distribution patterns, weight fraction, and geometric parameters of GPLs, as well as different types of AVLs, on the size-dependent buckling load and natural frequency are scrutinized in detail. The results show that the synchronized axial distributions of GPLs and AVLs could improve the buckling resistance and natural frequency more powerfully.

## 1. Introduction

Due to the excellent physical properties of graphene, graphene-based nano-reinforcements are identified as a kind of promising candidate for the reinforcement phases of polymer matrix nanocomposites [1]. An increasing number of scientists are working to apply them to the design of micro/nanoscale devices, including nanosensors, nanoactuators, nanotransducers, and biosensors [2,3,4,5]. Of all of the graphene-based nanofillers, graphene nanoplates (GPLs), with their high specific surface area and high surface-to-mass ratio, provide several special advantages to develop high performance advanced nanocomposites with a wide range of possible applications [6,7]. The mechanical performances of nanocomposites can be substantially improved by dispersing a low content of GPLs. The elastic modulus and tensile strength of the pure epoxy can be improved by 31% and 53%, respectively, through adding GPLs [8]. In addition, the benefits of GPLs are critically dependent on the GPL distribution pattern within the matrix. Under the development of functionally graded materials (FGMs), functionally graded GPL-reinforced nanocomposites (FG-GPLRCs) are created as an exclusive category of advanced non-homogeneous nanocomposites [9,10]. Kitipornchai et al. [11] carried out an analysis of the elastic buckling and free vibration of FG-GPLRC porous beams. Feng et al. [9] studied the nonlinear bending of non-uniformly distributed GPL-reinforced polymer nanocomposite beams. Wu et al. [12] performed the dynamic stability analysis of FG-GPLRC beams. Yang et al. [13] analyzed the buckling/postbuckling of FG-GPLRC multilayer beams. In addition, the static and dynamic characteristics of FG-GPLRC plate- [14,15], shell- [16,17], and arche-like [18,19] structures were examined carefully.

Upon reviewing the aforementioned research, there has been a significant focus on incorporating GPL content grading in the structural thickness direction. However, axially functionally graded (AFG) beams are extensively used in a variety of engineering fields, e.g., the automotive industry, helicopter rotor blades, MEMS, and turbine blades. The AFG-GPLRCs will allow a novel tailored fit to control the mechanical responses of nanocomposite structures, such as the buckling resistance and dynamic behaviors over a pre-specified level. It can be anticipated that the combination of both axial and thickness directions for beam-like structures will yield the best functional grading. Hein and Feklistova [20] showed the results of a free vibration of the AFGM beam using the method of Haar wavelets. Rokni et al. [21] focused on the optimal multi-walled carbon nanotubes (MWCNTs) distribution within a polymer composite microbeam to maximize its dynamic behaviors while using a fixed amount of MWCNTs. Their findings revealed that a non-uniform axial dispersion of MWCNTs resulted in higher natural frequencies compared to that of a uniform distribution pattern. This suggests that carefully controlling the dispersion pattern of MWCNTs can significantly enhance the performance of microbeams in terms of their vibrational characteristics. El-Ashmawy and Xu [22] demonstrated that the axially graded CNTs led to notable improvements in mechanical properties such as stiffness, strength, and durability. Rezaiee-Pajand et al. [23] introduced the Hencky bar-chain model to investigate the buckling behavior of AFG-composite beams, considering the axially graded distribution of carbon nanotubes. It should be noted that, the analyses for AFG beams have become more complicated because of the governing equation with variable coefficients, and great efforts have been made to try to solve the four-order differential equations as mentioned above. Liang et al. [24] established linear and nonlinear isogeometric finite element models of an AFG-GPLRC curved beam within the framework of the third-order shear deformation beam theory and von-Karman’s nonlinear geometric relation. Recently, Liu et al. [25] developed the state-space method to examine the impact of an axially varying dispersion of GPLs on the stability and dynamic behaviors of AFG-GPLRC beams subjected to edge (compressive) loads.

Thin beams have indeed found significant applications in micro-electro-mechanical systems (MEMS), such as those in vibration shock sensors, biosensors, etc. In those applications, the beam mostly falls within the size of microns and sub-microns, and it is common to observe a size-dependent behavior in the deformation [26]. Nateghi et al. [27] revealed that the size dependency of FG microbeams is much higher than that of macro-beams. Wang et al. [28] employed the modified couple stress theory (MCST) and the von Karman nonlinearity for the vibration analysis of microbeams. Allahkarami and Tohidi [29] applied MCST into checking the geometrically nonlinear vibration of multilayer FG-GPLRC microbeam. Yin et al. [30] put forward an analytical solution and employed an isogeometric approach for a comprehensive investigation of the buckling analysis of size-dependent microbeams. Nguyen et al. [31] developed a Chebyshev–Ritz solution to investigate bending, vibration, and buckling responses of porous microbeams. Soltani et al. [32] investigated the comprehensive study of the vibration control of multi-layer sandwich composite piezoelectric microbeams.

Indeed, most of the buckling studies on beams often consider the presence of end compressive loads. However, in real-world scenarios, beams often experience axially varying compressive loads along the beam length. This variation in compressive load can be due to factors such as distributed loads, bending moments, or other external forces acting on the beam. Karamanli and Aydogdu [33] focused on studying the elastic buckling behavior of beams made of isotropic materials, laminated composites, and sandwich structures subjected to various axially varying in-plane loads. Eltaher et al. [34] used a differential quadrature method (DQM) to examine the static stability and mode-shapes of axially varying in-plane loaded composite laminated beams. Masjedi and Weaver [35] derived an analytical solution for three-dimensional static deflection of composite beams that experience non-uniformly distributed axial loads. Howaon and Williams [36] presented a dynamic stiffness matrix analysis on the vibration of a beam-column with axially loaded Timoshenko members. Naguleswaran [37] examined the transverse vibration of beams under linearly varying axial force. Bargozini et al. [38] studied the critical buckling load for a sandwich composite beam reinforced with carbon nanorods in the bottom and top layers under variable axial forces based on the sinusoidal shear deformation theory.

As indicated by the literature review, there has been a significant amount of research dedicated to FG-GPLRC structures. However, based on the available literature, it appears that the static and dynamic behaviors of AFG-GPLRC microbeams subjected to axially varying loads have not been extensively studied or clearly addressed. For axially graded microbeams, similar problems become more complicated because of the governing equation with variable coefficients, which is quite difficult to solve analytically or numerically. So far, few solutions are found for arbitrary gradient change due to the difficulty of the mathematical treatment of the problem, save certain special gradients. The objective of the present paper is to discuss the buckling and vibration characteristics of axially functionally graded (AFG) graphene platelets reinforced (GPLRC) nanocomposite microbeams subjected to different types of axially varying loads (AVLs). The GPL nano-reinforcements are dispersed into an epoxy matrix throughout the beam length in the uniform and non-uniform patterns, while the non-uniform distributions of GPLs yield the axially functionally graded nanocomposites. In the meantime, the linear and nonlinear forms of AVLs are taken into consideration. By combining the improved Halpin–Tsai model and the rule of mixtures, the effective material properties of AFG-GPLRCs are calculated. The governing equations are derived on the assumptions of the Euler–Bernoulli beam theory, and the MSCT is adopted to capture the scale effect of microbeams. The state-space method is developed to derive the governing equation of the eigenvalue problems, and a first-order differential equation in matrix form is obtained. To solve the governing equation with variable coefficients, the length-direction discrete model composed of equilong segmentations is employed. The characteristic equations are finally established for various boundary conditions by using the continuous conditions of the fictitious interfaces of the discrete model, and are solved numerically. To examine the synergetic influences of the axial grading of GPLs and AVLs on the size-dependence buckling and dynamic characteristics, numerical studies are carried out in terms of the small scale, distribution patterns, and geometric parameters of GPLs, and various types of AVLs.

## 2. Theoretical Formulation

### 2.1. Modeling of AFG-GPLRCs

**Figure 1:**
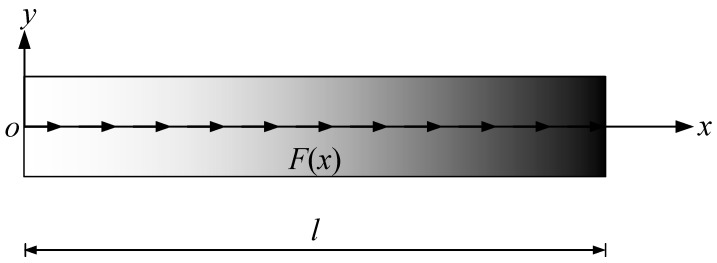
AFG-GPLRC microbeams under axially varying loads.

**Figure 2:**
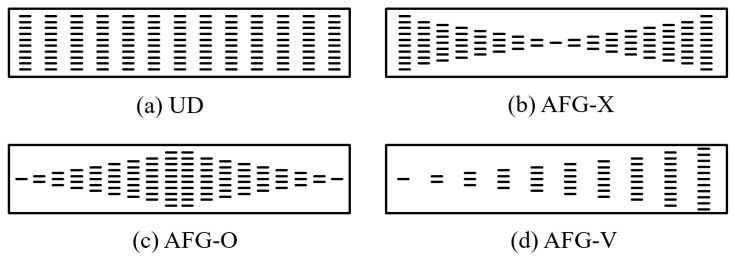
Schematic views of axial distribution patterns of GPLs.

An AFG-GPLRC microbeam (thickness h, length l, and width b) subjected to axially varying load F(x) is presented in Figure 1. The origin of the orthogonal coordinate system xoy is placed at the left-end cross-sectional center of the microbeam. The AFG-GPLRC microbeam is made from a matrix of polymer, and GPL nano-reinforcements are dispersed throughout the beam length direction in uniform or non-uniform manners with identical amounts. The axial distribution patterns of GPLs herein are shown in Figure 2.

The volume fraction $V_{\mathrm{GPL}}$ of GPLs for different distribution patterns, which varies along the beam length, can be addressed as [25]

(1) $$\begin{array}{ll} \mathrm{UD}: & V_{\mathrm{GPL}}\left(x\right)=V_{\mathrm{GPL}}^{*}\\ \mathrm{AFG}-X: & V_{\mathrm{GPL}}\left(x\right)=V_{\mathrm{GPL}}^{*}4\left|x-l/2\right|/l\\ \mathrm{AFG}-O: & V_{\mathrm{GPL}}\left(x\right)=V_{\mathrm{GPL}}^{*}\left(2-4\left|x-l/2\right|/l\right)\\ \mathrm{AFG}-V: & V_{\mathrm{GPL}}\left(x\right)=V_{\mathrm{GPL}}^{*}\left(1+2\left(x-l/2\right)/l\right) \end{array}$$

where $V_{\mathrm{GPL}}^{*}$ is the average GPL volume fraction, and can be evaluated from the weight fraction *W*_GPL_ as

(2) $$V_{\mathrm{GPL}}^{*}=\frac{W_{\mathrm{GPL}}}{W_{\mathrm{GPL}}+\left(\rho_{\mathrm{GPL}}/\rho_{M}\right)\left(1-W_{\mathrm{GPL}}\right)}$$

in which $W_{\mathrm{GPL}}$ is the total GPL weight fraction, and $\rho_{\mathrm{GPL}}$ and $\rho_{M}$ denote, respectively, the mass densities of GPLs and the matrix. It can be observed that the GPL volume fraction remains constant along the length direction in the UD pattern. For AFG-X, both of the ends are rich in GPL content, while the middle section has a lower GPL volume fraction. However, the GPL content in AFG-O is opposite to AFG-X. The GPL content of AFG-V represents a monotonous increase from the left end to the right end of the microbeam.

Herein, the multi-scaled model approximates the elastic modulus of GPLRCs, which is calculated based on the improved Halpin–Tsai micromechanics model [14], as follows:

(3) $$E=\frac{3}{8}\frac{1+\xi_{l}\eta_{l}V_{\mathrm{GPL}}}{1-\eta_{l}V_{\mathrm{GPL}}}\times E_{M}+\frac{5}{8}\frac{1+\xi_{w}\eta_{w}V_{\mathrm{GPL}}}{1-\eta_{w}V_{\mathrm{GPL}}}\times E_{M}$$

where $E_{\mathrm{GPL}}$ and $E_{M}$ denote, respectively, the elastic moduli of GPLs and polymer matrix. The parameters $\xi_{l}$ and $\xi_{w}$ read as

(4) $$\begin{array}{l} \eta_{l}=\frac{\left(E_{\mathrm{GPL}}/E_{M}\right)-1}{\left(E_{\mathrm{GPL}}/E_{M}\right)+\xi_{l}},\;\;\eta_{w}=\frac{\left(E_{\mathrm{GPL}}/E_{M}\right)-1}{\left(E_{\mathrm{GPL}}/E_{M}\right)+\xi_{w}}\\ \xi_{l}=2\frac{l_{\mathrm{GPL}}}{h_{\mathrm{GPL}}},\;\;\xi_{w}=2\frac{w_{\mathrm{GPL}}}{h_{\mathrm{GPL}}} \end{array}$$

where $l_{\mathrm{GPL}}$, $w_{\mathrm{GPL}}$ and $h_{\mathrm{GPL}}$ are the length, width, and thickness of GPLs, respectively.

Furthermore, the equivalent mass density ρ and Poisson’s ratio ν of GPLRCs are determined by using the mixture rule as in [14]

(5) $$\begin{array}{l} \rho =\rho_{\mathrm{GPL}}V_{\mathrm{GPL}}+\rho_{M}\left(1-V_{\mathrm{GPL}}\right)\\ \nu =\nu_{\mathrm{GPL}}V_{\mathrm{GPL}}+\nu_{M}\left(1-V_{\mathrm{GPL}}\right) \end{array}$$

in which $\nu_{\mathrm{GPL}}$ and $\nu_{M}$ are the Poisson’s ratios of GPLs and polymer matrix, respectively.

### 2.2. Axially Varying Load

In the present study, various types of load profiles along the beam length are considered and can be represented by a function as

(6) $$F\left(x\right)=F_{0}g\left(x\right)=F_{0}\left(\alpha_{0}+\alpha_{1}\frac{x}{l}+\alpha_{2}\frac{x^{2}}{l^{2}}\right)$$

where $F_{0}$ denotes the average value of the applied load, and the different values of $\alpha_{i=0,1,2}$ are given in Table 1. It is noted that the integrals of *F*(*x*) along the length of the microbeam are equal for all types of axially varying load cases.

**Figure 3:**
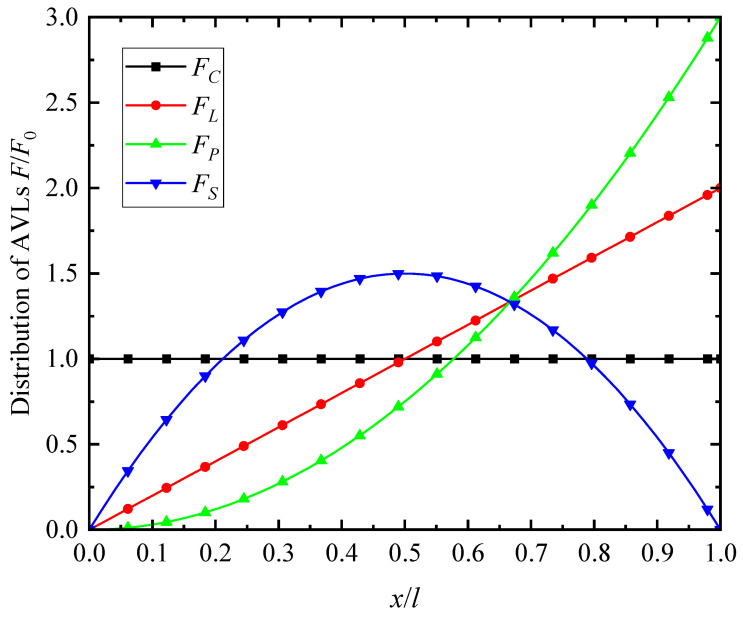
Distribution of the AVLs along the microbeam length.

Figure 3 shows the distribution of the different types of AVLs throughout the length of the microbeam. As can be observed, *F_C_* is the constant-load case; *F_L_* monotonically increases from the left to the right end; *F_P_* varies in form of parabolic variation; and *F_S_* varies as a parabolic form.

### 2.3. Governing Equations

**Figure 4:**
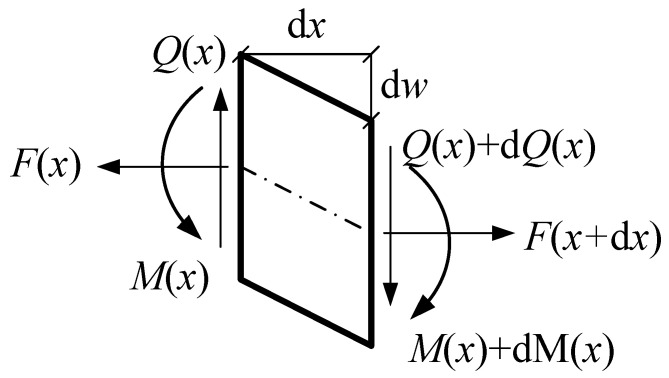
Differential element of the microbeam.

The equilibrium of differential elements in the microbeam subject to the axial load *F* is shown in Figure 4, and the following relationships must be valid

(7) $$\frac{\partial Q}{\partial x}=\rho A\frac{\partial^{2}w}{\partial t^{2}}$$

(8) $$\frac{\partial M}{\partial x}=F\left(x\right)\frac{\partial w}{\partial x}-Q\left(x\right)$$

where *Q*(*x*) is the shear force; *w*(*x*) is deflection; and *M*(*x*) is named as the equivalent moment combining the bending and couple moments, and is written as [25]

(9) $$M=M_{x}+M_{xy}=\left(EI+\zeta^{2}GA\right)\frac{\partial^{2}w}{\partial x^{2}}=\bar{E}I\frac{\partial^{2}w}{\partial x^{2}}$$

in which *A* is the cross-sectional area, *I* is the inertia moment, *M_x_*(*x*) is the bending moment, and *M_xy_*(*x*) is the couple moment induced by couple stresses. Herein $\bar{E}=E+\frac{\zeta^{2}GA}{I}=E+12G\frac{\zeta^{2}}{h^{2}}$ is the size-dependent equivalent Young’s modulus of the microbeam, and can be decided both by the material length scale and geometric parameters of the microbeam.

The microbeam cross-sectional slope can be introduced as

(10) $$\varphi =\frac{\partial w}{\partial x}$$

Then, Equations (8) and (9) are to be rewritten as

(11) $$\frac{\partial M}{\partial x}=F\left(x\right)\varphi \left(x\right)-Q\left(x\right)$$

(12) $$\frac{\partial \varphi }{\partial x}=\frac{M}{\bar{E}I}$$

Equations (7) and (10)–(12) can be rewritten as a matrix form

(13) $$\frac{dT\left(x\right)}{dx}=B\left(x\right)T\left(x\right)$$

where the so-called state vector is $T\left(x\right)={\left[\begin{array}{cccc} w\left(x\right) & \varphi \left(x\right) & M\left(x\right) & Q\left(x\right) \end{array}\right]}^{T}$, and the system matrix **B**(*x*) leads to

(14) $$B\left(x\right)=\left[\begin{array}{cccc} 0 & 1 & 0 & 0\\ 0 & 0 & \frac{1}{E\left(x\right)I} & 0\\ 0 & F\left(x\right) & 0 & -1\\ \rho \left(x\right)A\frac{\partial^{2}}{\partial t^{2}} & 0 & 0 & 0 \end{array}\right]$$

Furthermore, the following parameters in dimensionless form are introduced

(15) $$\xi =\frac{x}{l},\;\;\bar{w}\left(\xi \right)=\frac{w\left(x\right)}{h},\;\;\bar{M}\left(\xi \right)=\frac{M\left(x\right)l}{E_{0}I},\;\;\bar{Q}\left(\xi \right)=\frac{Q\left(x\right)l^{2}}{E_{0}I},\;\;\Omega =\omega l^{2}\sqrt{\frac{\rho_{0}A}{E_{0}I}},\;\;{\bar{F}}_{0}=\frac{F_{0}l^{2}}{E_{0}I}$$

For the problem of vibration, the state vector is

(16) $$T\left(\xi ,t\right)=\bar{T}\left(\xi \right)e^{i\omega t}$$

and Equation (13) turns out to be

(17) $$\frac{d\bar{T}\left(\xi \right)}{d\xi }=\bar{B}\left(\xi \right)\bar{T}\left(\xi \right)$$

where $\bar{T}\left(\xi \right)={\left[\begin{array}{cccc} \bar{w}\left(\xi \right) & \varphi \left(\xi \right) & \bar{M}\left(\xi \right) & \bar{Q}\left(\xi \right) \end{array}\right]}^{T}$, and the updated system matrix **B**(*x*) is

(18) $$\bar{B}\left(\xi \right)=\left[\begin{array}{cccc} 0 & \frac{l}{h} & 0 & 0\\ 0 & 0 & \frac{E_{0}}{\bar{E}\left(\xi \right)} & 0\\ 0 & {\bar{F}}_{0}g\left(\xi \right) & 0 & -1\\ -\frac{h}{l}\frac{\rho \left(\xi \right)}{\rho_{0}}\Omega^{2} & 0 & 0 & 0 \end{array}\right]$$

in which, respectively, $E_{0}$ and $\rho_{0}$ are the referenced values, and $g\left(\xi \right)=\alpha_{0}+\alpha_{1}\xi +\alpha_{2}\xi^{2}$ represents the distribution of AVLs throughout the microbeam length.

## 3. Solution Procedure

**Figure 5:**
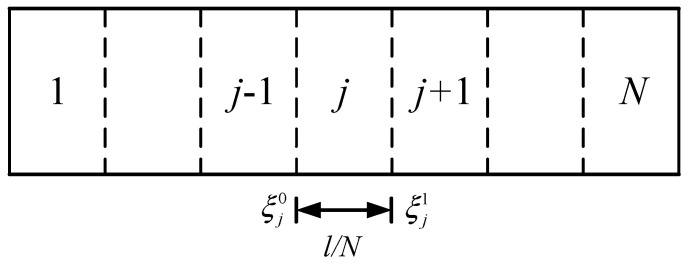
Axially discrete model of AFG-GPLRC microbeams.

The governing Equation (17) is a variable-coefficient ordinary differential equation, and is quite difficult to solve analytically. To facilitate the numerical calculations, the microbeam is split into a series of identical length segments $l_{e}=l/N$ along its length direction with a large enough value of *N,* as shown in Figure 5. The material properties of each sufficiently short segment are thought of as constant, resulting in matrix $\bar{B}$ independent of *ξ*.

For the *j*th segment, the governing equation, Equation (17), is

(19) $$\frac{d{\bar{T}}_{j}\left(\xi \right)}{d\xi }={\bar{B}}_{j}\left(\xi_{j_{m}}\right){\bar{T}}_{j}\left(\xi \right)$$

where $\xi_{j}^{0}=\left(j-1\right)/N$ and $\xi_{j}^{1}=j/N$ represent contiguous coordinates of the segment, and $\xi_{j_{m}}=\left(\xi_{j}^{0}+\xi_{j}^{1}\right)/2,\;\;\left(j=1,2,\cdots ,N\right)$. The solution for Equation (19) is

(20) $${\bar{T}}_{j}\left(\xi \right)=\exp\left[\left(\xi -\xi_{j}^{0}\right){\bar{B}}_{j}\right]{\bar{T}}_{j}\left(\xi_{j}^{0}\right),\;\;\left(\xi_{j}^{0}\leq \xi \leq \xi_{j}^{1}\right)$$

Setting $\xi =\;\xi_{j}^{1}$ leads to

(21) $${\bar{T}}_{j}\left(\xi_{j}^{1}\right)=\exp\left({\bar{B}}_{j}/N\right){\bar{T}}_{j}\left(\xi_{j}^{0}\right)$$

The continuity conditions of adjacent segments yield

(22) $$\bar{T}\left(1\right)=R\bar{T}\left(0\right)$$

in which $\bar{T}\left(0\right)={\bar{T}}_{1}\left(\xi_{1}^{0}\right)$ and $\bar{T}\left(1\right)={\bar{T}}_{N}\left(\xi_{N}^{1}\right)$ are the state vectors of the microbeam’s two ends, respectively, and $R=\prod_{j=N}^{1}\exp\left({\bar{B}}_{j}/N\right)$ denotes the microbeam transfer matrix.

Three typical end boundary conditions of the microbeams, namely simply supported (S), clamped (C), and free (F) ends, are herein considered as

$\bar{w}=0,\;\;\bar{M}=0$, simply supported end;

$\bar{w}=0,\;\;\varphi =0$, clamped end;

$\bar{M}=0,\;\;\bar{Q}=0$, free end.

For the SS microbeam, Equation (22) gives

(23) $$\left\{\begin{array}{c} 0\\ \varphi \left(1\right)\\ 0\\ \bar{Q}\left(1\right) \end{array}\right\}=\left[\begin{array}{cccc} R_{11} & R_{12} & R_{13} & R_{14}\\ R_{21} & R_{22} & R_{23} & R_{24}\\ R_{31} & R_{32} & R_{33} & R_{34}\\ R_{41} & R_{42} & R_{43} & R_{44} \end{array}\right]\left\{\begin{array}{c} 0\\ \varphi \left(0\right)\\ 0\\ \bar{Q}\left(0\right) \end{array}\right\}$$

in which $R_{ij}$ is the element of the global transfer matrix **R**. The non-zero solution for the first and third equations in Equation (23) requires

(24) $$\left|\begin{array}{cc} R_{12} & R_{14}\\ R_{32} & R_{34} \end{array}\right|=0$$

which is the characteristic equation of SS microbeams.

Similarly, the corresponding characteristic equation for the CC microbeam is

(25) $$\left|\begin{array}{cc} R_{13} & R_{14}\\ R_{23} & R_{24} \end{array}\right|=0$$

The characteristic equation for the CF microbeam gives

(26) $$\left|\begin{array}{cc} R_{33} & R_{34}\\ R_{43} & R_{44} \end{array}\right|=0$$

The CS microbeam yields

(27) $$\left|\begin{array}{cc} R_{13} & R_{14}\\ R_{33} & R_{34} \end{array}\right|=0$$

Equations (24)–(27) show two eigenvalue problems which lead to the calculation of vibration frequencies and buckling loads for the AFG-PLRC microbeams, and can be solved with numerical methods.

## 4. Numerical Results

In this section, the present formulations are applied herein to examine the stability and vibration characteristics of AFG-GPLRC microbeams. Firstly, numerical examples are carried out to validate the accuracy and convergence of the present method. Then the buckling load and vibration frequency of AFG-GPLRC microbeams with AVLs are studied. In the calculations, the parameters in Table 2 are used.

Unless otherwise indicated, the geometrical parameters of GPL nano-reinforcements are fixed as: $l_{\mathrm{GPL}}=2.5\;\mu m$, $w_{\mathrm{GPL}}=1.5\;\mu m$, and $h_{\mathrm{GPL}}=1.5\;\mathrm{nm}$, respectively. The slenderness ratio λ=l/h of the microbeam is set to be λ=20, and the GPL weight fraction is preferred as *W*_GPL_ = 1%. The number *N* of segments is taken as 200 to maintain sufficient convergence.

Equations (24)–(27) are transcendental equations about the nondimensional vibration frequency Ω, and the critical buckling loads can be calculated by setting Ω = 0 for the corresponding boundary conditions. In the present study, the bisection method with the aid of Matlab R2022a is conducted to find the roots of the transcendental equation.

### 4.1. Validation

Furthermore, to validate the present model, a unit-length beam consisting of AFGMs without considering size effects, i.e., ζ=0, are examined. The Young’s modulus *E* of the AFGM beam [37] takes the form of $E\left(x\right)=E_{0}\left(1+x\right)$, while the mass density *ρ* is chosen as $\rho \left(x\right)=\rho_{0}\left(1+x+x^{2}\right)$. Table 3 lists the parameters for the frequency $\mu =\omega l^{2}\sqrt{\rho_{0}A/E_{0}I}$ of the AFGM beams, and compares these to the existing results from the literature. It shows good agreement between our results and the available previous results.

The validations of the free vibration and buckling load calculations of a linearly axial-loaded isotropic beam were also conducted. The vibration frequency and buckling parameters predicted by the proposed model match well, as shown in Table 4 and Table 5, in which the parameters in the tables can be found in Ref. [36].

### 4.2. Buckling Analysis of AFG-GPLRC Microbeams

The influence of the material length scale, axial distribution pattern of GPLs, types of axially varying load, and boundary condition on the critical buckling load ${\bar{F}}_{\mathrm{cr}}=F_{\mathrm{cr}}l^{2}/E_{0}I$ of AFG-GPLRC microbeams are presented in Table 6, Table 7, Table 8 and Table 9. For comparison, the results of pure epoxy microbeams are also given. It is discovered that the buckling resistance of the nanocomposite microbeams could be greatly increased by a low percentage of GPL nanofillers. With a dispersion of 1% weight fraction of GPLs in AFG-O SS microbeams, it is over five times higher than the buckling load of a pure-epoxy case. This is because the presence of GPL nanofillers enhances the effective material properties, resulting in increased flexural beam stiffness. Additionally, the axially graded pattern of GPLs significantly influences the buckling resistance. The table results show that GPL nanofillers have a significant enhancing impact on various types of axial load distributions. Among the studied GPL distributions, the AFG-V pattern for CC beams reaches a higher buckling load for the symmetric parabolic load, in which the axial distributions of GPLs and the applied load are nearly coincident along beam length. Among all of the axially varying loads, the AFG-O SS microbeams exhibit the highest buckling resistance, except for in the parabolic case. For the same GPL patterns, the buckling load increases as $F_{P}<F_{L}<F_{C}<F_{S}$. It is as a result of this that, for the symmetric parabolic axial load, the load magnitude is located at the min-span of the microbeam, and the GPL content is also higher at the same position for the AFG-O pattern. This increased stiffness helps to resist buckling under the higher load magnitude at the mid-span of the microbeam, resulting in improved buckling resistance for the AFG-O pattern in this specific case. However, the CC and CF microbeams with the pattern UD under the axial constant load $F_{C}$ yield the highest buckling load. The effects of the material length scale parameter on the buckling loads of the AFG-GPLRC microbeams are also listed in Table 9, Table 10, Table 11 and Table 12. As can be observed, the critical buckling loads by the MCST are significantly different with the classical ones (*ζ*/*h* = 0), and the buckling resistance of the microbeam with size effects increases as the material length scale parameter takes higher values for all of the studied cases. As expected, the CC boundary conditions yield the highest buckling load for a given GPL pattern and axially applied load. It can be concluded that the synergetic influence of the nature of the axial gradation of GPLs, axially applied load, and material length scale, as well as the boundary condition, on the buckling resistance of the AFG-GPLRC microbeam are intricate. Generally, the synchronized axial distributions of GPLs and applied load throughout the beam length could improve the buckling resistance more powerfully.

**Figure 6:**
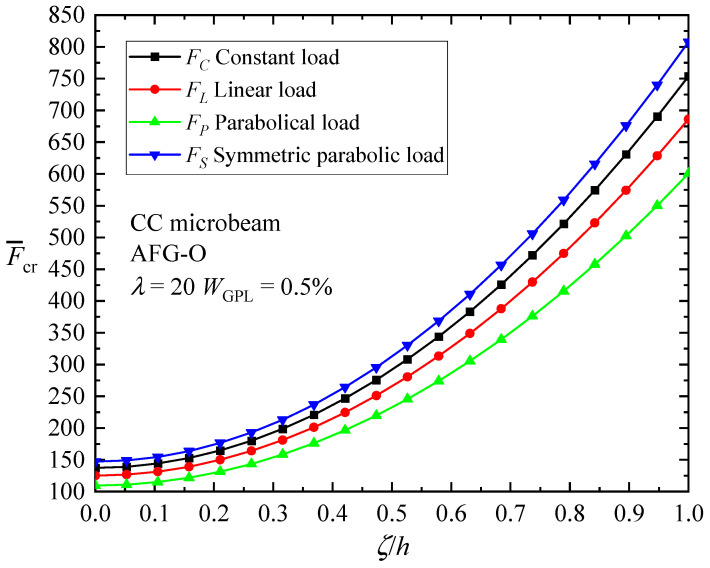
Size effect on the critical buckling load for AFG-GPLRC CC microbeams under AVLs.

To further examine the size effect of AFG-GPLRC microbeams, the critical buckling loads with respect to material length scale parameter to thickness ratio ζ/h for CC microbeams are presented in Figure 6. It is observed again that the material length scale enhances the buckling load, i.e., the intrinsic size dependence of the microbeam increases the bending stiffness, leading to increased values of critical buckling load, which confirms the stiffening effect of the length scale parameter.

**Figure 7:**
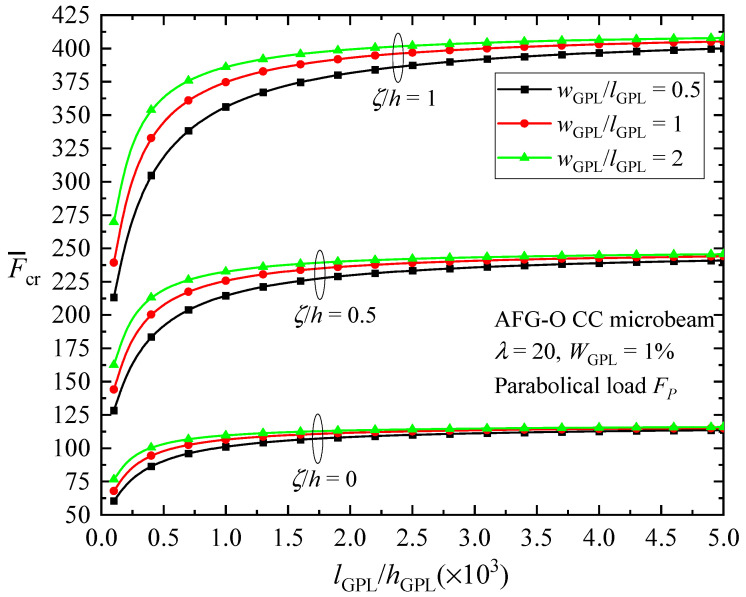
Effect of GPL geometry parameter on the buckling load of CC AFG-O microbeams with respect to different material length scale parameters.

Figure 7 plots the change in the critical buckling load of the AFG-O CC beam with respect to GPL geometry parameters, considering the change in the length-to-thickness ratio $l_{\mathrm{GPL}}/h_{\mathrm{GPL}}$ and aspect ratio $w_{\mathrm{GPL}}/l_{\mathrm{GPL}}$ of GPLs, as well as in the material length scale parameter ζ/h. The length of GPLs is held constant ($l_{\mathrm{GPL}}=2.5\;\mu m$) in the figure. The buckling load firstly increases quickly as $l_{\mathrm{GPL}}/h_{\mathrm{GPL}}$ increases, and then increases slowly for higher $l_{\mathrm{GPL}}/h_{\mathrm{GPL}}$ ratios. It is concluded that nanofillers consisting of a smaller number of monolayer graphene sheets are expected to provide superior enhancing effects. In the meantime, it is seen that the increase in the width of GPLs leads to a rise in the value of the critical buckling load, which indicates that an increase in the area of the GPLs leads to a higher bending stiffness of the GPL-reinforced microbeams. Figure 7 shows again the hardening effect of a micro-scaled beam due to the intrinsic material length.

**Figure 8:**
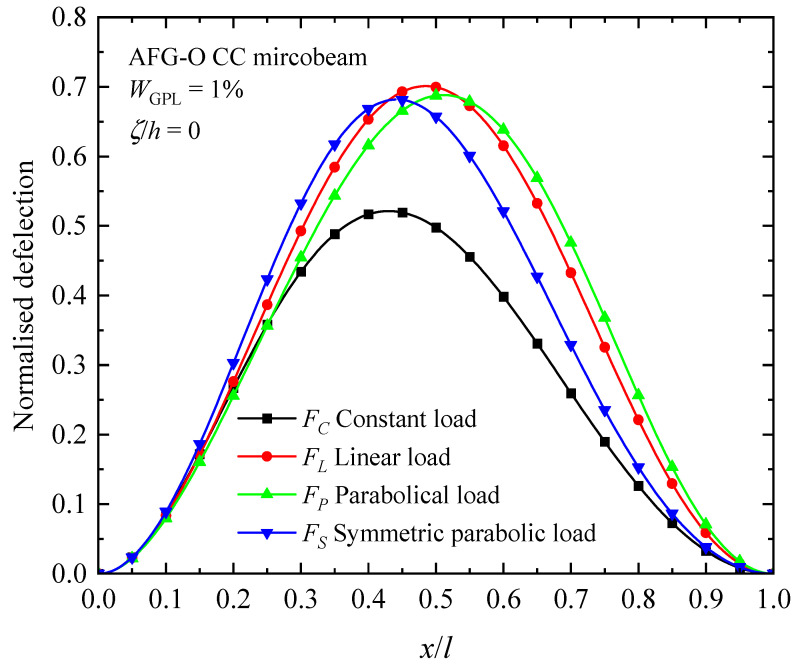
Buckling mode shapes of the AFG-GPLRC microbeam with respect to various axially varying loads.

**Figure 9:**
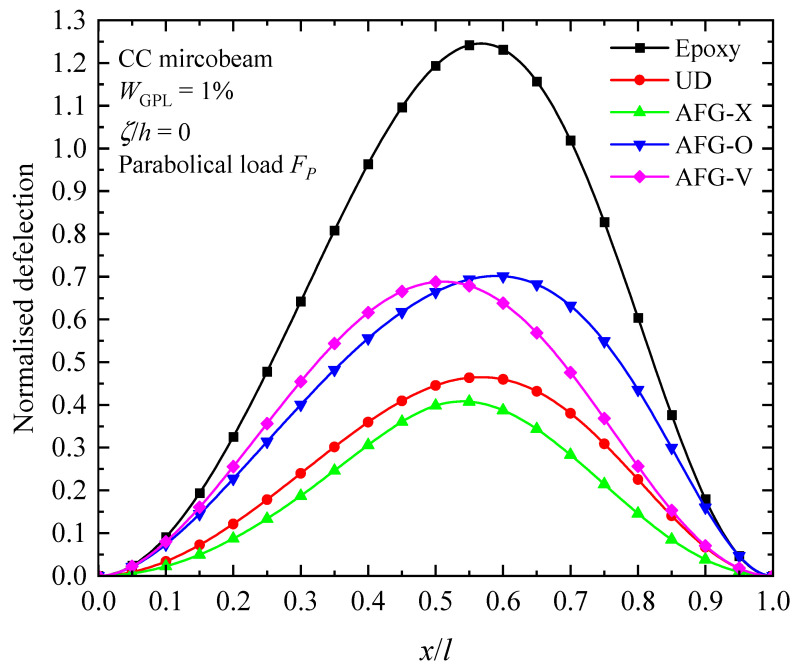
Buckling mode shapes of the AFG-GPLRC microbeam with respect to various axial GPL distribution patterns.

**Figure 10:**
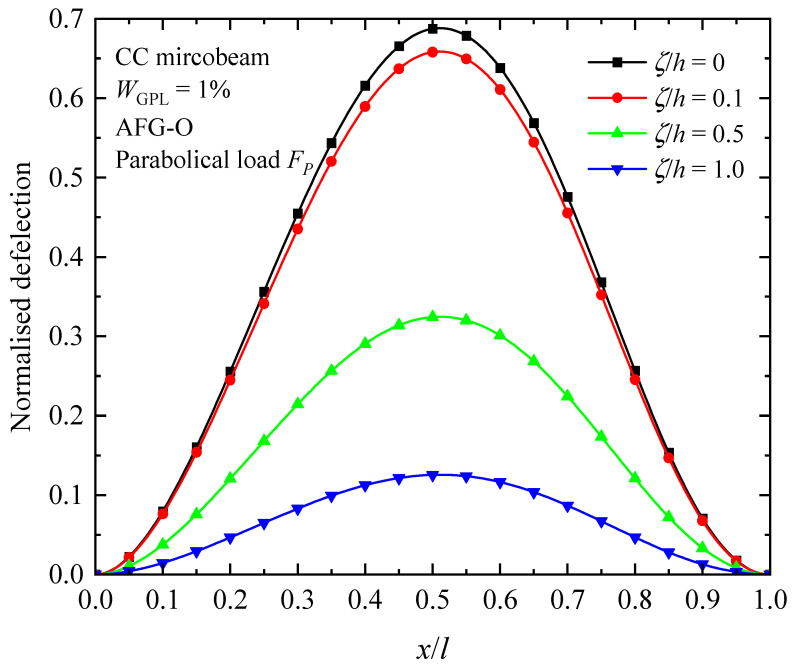
Buckling mode shapes of the AFG-GPLRC microbeam with respect to various material length scale parameters.

The influence of the nature of axially varying loads, axially graded dispersion of GPL nanofillers, and material intrinsic length scale on the fundamental buckling mode of the AFG-GPLRC CC microbeam are presented in Figure 8, Figure 9 and Figure 10. From the figures, the buckling mode shapes of the microbeam under an axially applied load is asymmetric along the microbeam length in spite of symmetric boundary condition, symmetric GPL dispersion pattern and symmetric axially applied load. This can be interpreted due to the accumulation of the axially applied load. Meantime, it can be found that the distribution of axial load has a critical impact on the shape of buckling mode. For a given axially varying load, the buckling mode varies sensitively with axial distribution pattern of GPLs, as seen in Figure 9. The GPL nano-reinforcements dispersed into epoxy matrix can observably improve the bending stiffness, and hence decreases the transverse deflection of AFG-GPLRC microbeams. For the given axially applied load, the AFG-X pattern holds the lowest peak value in the buckling mode. From Figure 10, it demonstrates that the transverse deflection of AFG-GPLRC microbeams decreases with the increase in the material length scale parameter. This means that the microbeam having a larger stiffness when considering size effect has a less transverse displacement.

### 4.3. Free Vibration of AFG-GPLRC Microbeams

To investigate the vibration behaviors of AFG-GPLRC microbeams, Table 10 lists the dimensionless fundamental frequency parameter Ω involved in axial GPL distribution patterns, boundary conditions, and the material length scale parameter to thickness ratios. It is seen from the table that the microbeam frequency rises significantly by adding GPL nano-reinforcements regardless of the GPL distribution pattern. As can be expected, the dispersion of GPLs leads to an increase in the microbeam bending stiffness. Similar tendencies found in the buckling study also apply to the reinforcing effects of GPL nanofillers on microbeam vibration performances, which rely on the boundary condition. It is important to note that the frequency increment is determined by both the boundary condition and the GPL distribution pattern. In other words, these two factors work together to influence the increase in frequency. This observation highlights the interplay between the microbeam boundary conditions and the GPL distribution pattern, both of which contribute to the changes in vibration frequencies. For instance, the AFG-O pattern exhibits a more pronounced enhancement in the vibration frequency of SS microbeams. On the other hand, the UD pattern yields the highest frequencies for microbeams with other boundary conditions. This suggests that the choice of GPL distribution pattern can have a significant impact on the vibration frequencies of microbeams, with different patterns showing varying degrees of enhancement depending on the specific boundary conditions. The size-dependent fundamental frequencies of the microbeams are also listed in Table 13. It can be found that the frequencies increase monotonously as the material length scale parameter *ζ* increases. It is due to this that the increasing *ζ* yields a more powerful small-scale effect that makes the microbeams stiffer.

**Table 10:** Fundamental frequency Ω of the AFG-GPLRC microbeam without considering axial load.

B.C.	*ζ*/*h*	Epoxy	UD	AFG-X	AFG-O	AFG-V
SS	0	9.8696	20.6166	15.2374	22.6759	19.6099
	0.1	10.0881	21.0737	15.5750	23.1788	20.0446
	0.5	14.3683	30.0243	22.1860	33.0263	28.5569
	1.0	23.0991	48.2773	35.6698	53.1070	45.9167
CC	0	22.3733	46.7355	45.9273	38.9470	42.3031
	0.1	22.8687	47.7716	46.9459	39.8104	43.2410
	0.5	32.5714	68.0616	66.8905	56.7173	61.6064
	1.0	52.3631	109.4392	107.5613	91.1966	99.0593
CF	0	3.5160	7.3446	7.2080	5.6277	8.7530
	0.1	3.5939	7.5074	7.3678	5.7524	8.9472
	0.5	5.1187	10.6960	10.4969	8.1942	12.7495
	1.0	8.2290	17.1986	16.8782	13.1745	20.5026
CS	0	15.4182	32.2070	29.5869	29.9310	27.6900
	0.1	15.7596	32.9211	30.2429	30.5946	28.3038
	0.5	22.4461	46.9036	43.0883	43.5895	40.3227
	1.0	36.0852	75.4183	69.2839	70.0900	64.8343

**Figure 11:**
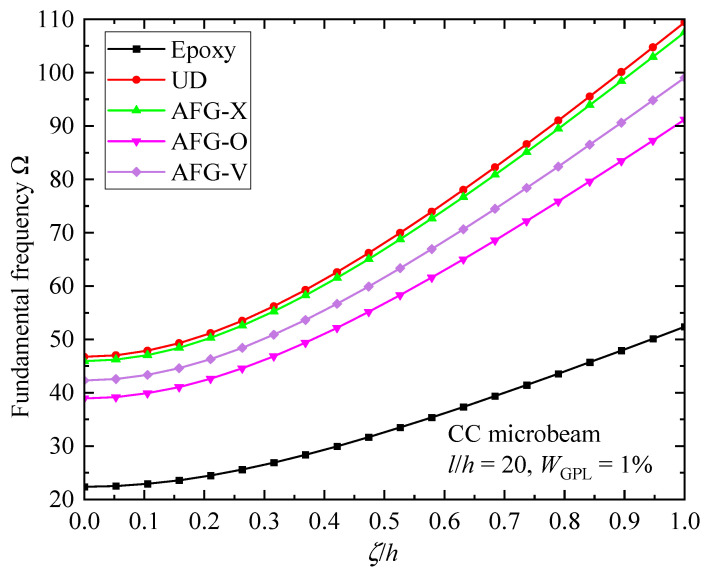
Size effects on the fundamental frequency Ω of the AFG-GPLRC CC microbeam.

To further illustrate the impacts of the GPL enhancement effect and micro-size effect, Figure 11 depicts the fundamental frequency parameter Ω of the AFG-GPLRC CC microbeams against the material length scale parameter. As can be observed, as the parameter *ζ* increases, the fundamental frequency consistently and monotonously increases. Compared with pure epoxy, the fundamental frequency of the AFG-GPLRC microbeam exhibits a sharper increase, which indicates that the GPL incremental effect on the fundamental frequency is strengthened by size effects. However, the incremental effect depends on the GPL distribution pattern. For the CC microbeams, the UD pattern produces the highest fundamental frequency, followed by AFG-X, AFG-V, and AFG-O patterns. It should, again, be pointed out that the enhancement of GPL nanofillers is determined by both the GPL distribution patterns and boundary conditions.

**Figure 12:**
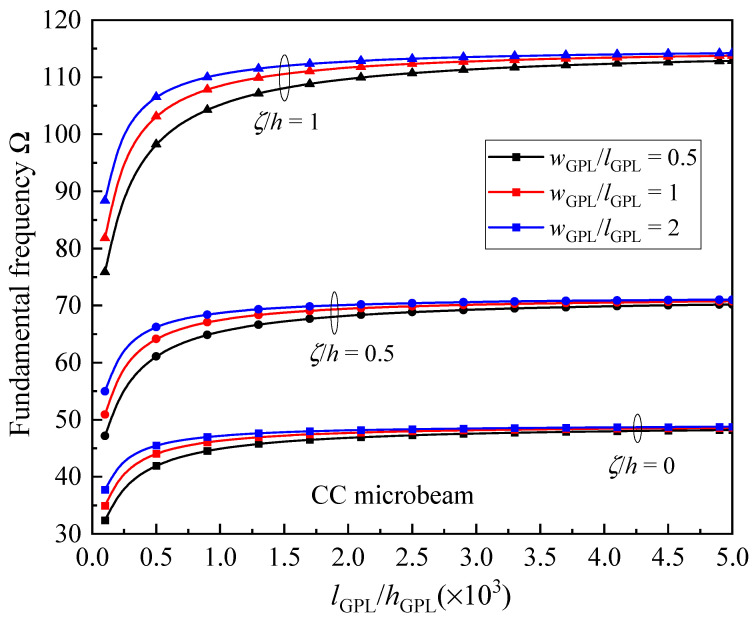
Effect of GPL geometry parameters on the fundamental frequency Ω of the CC microbeam.

Figure 12 studies the fundamental frequency variation versus the size and geometry of GPLs for the UD CC microbeams against different material length scale parameters. A constant GPL length *l*_GPL_ = 2.5 μm is kept during analysis. As shown in Figure 12, the frequency first increases very fast, and then slowly increases for larger *l*_GPL_/*h*_GPL_. It can be noted here that the GPL nano-reinforcements contain fewer graphene layers for a larger value of *l*_GPL_/*h*_GPL_, and hence improve the microbeam frequencies more effectively. It is also seen that the microbeams reinforced by GPLs with larger *w*_GPL_/*l*_GPL_ produce larger frequencies. This means that the larger surface area of GPL nano-reinforcements provided a more superior reinforcement effect.

Table 11, Table 12, Table 13 and Table 14 reveal the influence of axial variations in the GPL distribution pattern and applied load on the fundamental frequency Ω of the AFG-GPLRC microbeam, respectively. It is noted the material length scale effect is not considered in these tables, i.e., $\left(\zeta /h=0\right)$, and ${\bar{F}}_{\mathrm{cr}}^{E}$ denotes the buckling load of a pure epoxy microbeam. To maintain consistency with the previous discussion, the positive values of ${\bar{F}}_{0}$ denote compressive loads and the negative ones represent tensional loads. From the tables, the dimensionless natural frequency descends with an increase in axial compression force. On the other hand, it rises with an enlarging of the axial tension force. These trends highlight the influence of axially applied loads on the vibration of microbeams and provide insights into the relationship between axially applied loads and natural frequencies. However, the synergetic dependences of the fundamental frequency on the GPL distribution pattern, axially applied load, and boundary condition are quite complex. It has been observed that the AFG-O pattern consistently yields the highest frequency for all given axially applied loads. In contrast, the UD microbeam gives the largest value of frequency for CC and CS boundary conditions, while AFG-V is the most effective in enhancing the vibration frequency of CF microbeams. For a given GPL distribution pattern and average value of applied load ${\bar{F}}_{0}$, the type of axially applied load can also critically influence the vibration performance. For instance, the AFG-O SS microbeams under a symmetric parabolic load $F_{S}$ hold the highest natural frequency regardless of compressive or tensional load. However, the AFG-V CF microbeam under a parabolical load $F_{P}$ and constant load $F_{P}$ yields the highest ones for tensional and compressive loads, respectively.

**Table 11:** Dimensionless fundamental frequency Ω for the AFG-GPLRC SS microbeam (${\bar{F}}_{\mathrm{cr}}^{E}=7.7171$).

AVLs	${\bar{F}}_{0}$	Epoxy	UD	AFG-X	AFG-O	AFG-V
F_C_	$-0.8{\bar{F}}_{cr}^{E}$	12.5834	22.0467	17.1536	23.9985	21.1403
	$-0.2{\bar{F}}_{\mathrm{cr}}^{E}$	10.6133	20.9832	15.7385	23.0137	20.0037
	$0.2{\bar{F}}_{\mathrm{cr}}^{E}$	9.0651	20.2433	14.7192	22.3329	19.2078
	$0.8{\bar{F}}_{\mathrm{cr}}^{E}$	6.0396	19.0796	13.0410	21.2706	17.9466
F_L_	$-0.8{\bar{F}}_{\mathrm{cr}}^{E}$	12.5092	22.0361	17.1426	23.9839	21.3487
	$-0.2{\bar{F}}_{\mathrm{cr}}^{E}$	10.6073	20.9825	15.7377	23.0127	20.0633
	$0.2{\bar{F}}_{\mathrm{cr}}^{E}$	9.0577	20.2425	14.7184	22.3319	19.1422
	$0.8{\bar{F}}_{\mathrm{cr}}^{E}$	5.8383	19.0666	13.0257	21.2529	17.6418
F_P_	$-0.8{\bar{F}}_{\mathrm{cr}}^{E}$	12.7911	22.2372	17.3180	24.2204	21.6802
	$-0.2{\bar{F}}_{\mathrm{cr}}^{E}$	10.7110	21.0382	15.7883	23.0784	20.1579
	$0.2{\bar{F}}_{\mathrm{cr}}^{E}$	8.9153	20.1827	14.6620	22.2612	19.0381
	$0.8{\bar{F}}_{\mathrm{cr}}^{E}$	4.6089	18.7977	12.7545	20.9349	17.1521
F_S_	$-0.8{\bar{F}}_{\mathrm{cr}}^{E}$	11.7775	21.6072	16.7620	23.4757	20.6275
	$-0.2{\bar{F}}_{\mathrm{cr}}^{E}$	10.3850	20.8693	15.6343	22.8789	19.8699
	$0.2{\bar{F}}_{\mathrm{cr}}^{E}$	9.3214	20.3603	14.8286	22.4706	19.3458
	$0.8{\bar{F}}_{\mathrm{cr}}^{E}$	7.4025	19.5682	13.5194	21.8411	18.5274

**Table 12:** Dimensionless fundamental frequency Ω for the AFG-GPLRC CC microbeam (${\bar{F}}_{\mathrm{cr}}^{E}=35.8365$).

AVLs	${\bar{F}}_{0}$	Epoxy	UD	AFG-X	AFG-O	AFG-V
F_C_	$-0.8{\bar{F}}_{\mathrm{cr}}^{E}$	29.1182	50.3587	50.1791	42.7745	46.3175
	$-0.2{\bar{F}}_{\mathrm{cr}}^{E}$	24.2563	47.6698	47.0344	39.9395	43.3458
	$0.2{\bar{F}}_{\mathrm{cr}}^{E}$	20.2974	45.7801	44.7869	37.9276	41.2308
	$0.8{\bar{F}}_{\mathrm{cr}}^{E}$	11.8556	42.7733	41.1347	34.6842	37.8099
F_L_	$-0.8{\bar{F}}_{\mathrm{cr}}^{E}$	28.9486	50.3328	50.1586	42.7218	46.9863
	$-0.2{\bar{F}}_{\mathrm{cr}}^{E}$	24.2417	47.6680	47.0330	39.9358	43.5401
	$0.2{\bar{F}}_{\mathrm{cr}}^{E}$	20.2780	45.7782	44.7854	37.9236	41.0141
	$0.8{\bar{F}}_{\mathrm{cr}}^{E}$	11.1889	42.7396	41.1075	34.6091	36.7696
F_P_	$-0.8{\bar{F}}_{\mathrm{cr}}^{E}$	28.7431	50.2761	49.8635	42.9003	47.3501
	$-0.2{\bar{F}}_{\mathrm{cr}}^{E}$	24.2113	47.6575	46.9548	39.9955	43.6537
	$0.2{\bar{F}}_{\mathrm{cr}}^{E}$	20.2810	45.7857	44.8671	37.8511	40.8807
	$0.8{\bar{F}}_{\mathrm{cr}}^{E}$	10.6588	42.7482	41.4612	34.2189	36.0722
F_S_	$-0.8{\bar{F}}_{\mathrm{cr}}^{E}$	28.9878	50.6885	50.6827	42.2585	46.1296
	$-0.2{\bar{F}}_{\mathrm{cr}}^{E}$	24.2701	47.6850	47.1846	39.8086	43.3033
	$0.2{\bar{F}}_{\mathrm{cr}}^{E}$	20.2281	45.7590	44.6167	38.0601	41.2699
	$0.8{\bar{F}}_{\mathrm{cr}}^{E}$	10.6099	42.6462	40.3016	35.2266	37.9410

**Table 13:** Dimensionless fundamental frequency Ω for the AFG-GPLRC CF microbeam (${\bar{F}}_{\mathrm{cr}}^{E}=1.4886$).

AVLs	${\bar{F}}_{0}$	Epoxy	UD	AFG-X	AFG-O	AFG-V
F_C_	$-0.8{\bar{F}}_{\mathrm{cr}}^{E}$	4.2112	7.7101	7.6807	6.0174	9.0995
	$-0.2{\bar{F}}_{\mathrm{cr}}^{E}$	3.7061	7.4381	7.3301	5.7280	8.8415
	$0.2{\bar{F}}_{\mathrm{cr}}^{E}$	3.3117	7.2495	7.0829	5.5253	8.6631
	$0.8{\bar{F}}_{\mathrm{cr}}^{E}$	2.5763	6.9541	6.6888	5.2038	8.3850
F_L_	$-0.8{\bar{F}}_{\mathrm{cr}}^{E}$	4.3737	7.8196	7.8649	6.0920	9.2236
	$-0.2{\bar{F}}_{\mathrm{cr}}^{E}$	3.7627	7.4680	7.3812	5.7490	8.8752
	$0.2{\bar{F}}_{\mathrm{cr}}^{E}$	3.2383	7.2178	7.0278	5.5026	8.6274
	$0.8{\bar{F}}_{\mathrm{cr}}^{E}$	2.0713	6.8150	6.4395	5.1006	8.2284
F_P_	$-0.8{\bar{F}}_{\mathrm{cr}}^{E}$	4.4156	7.8617	7.9465	6.1139	9.2781
	$-0.2{\bar{F}}_{\mathrm{cr}}^{E}$	3.7842	7.4803	7.4055	5.7560	8.8913
	$0.2{\bar{F}}_{\mathrm{cr}}^{E}$	3.2035	7.2040	7.0004	5.4942	8.6095
	$0.8{\bar{F}}_{\mathrm{cr}}^{E}$	1.7028	6.7496	6.3059	5.0579	8.1436
F_S_	$-0.8{\bar{F}}_{\mathrm{cr}}^{E}$	4.2511	7.7287	7.6887	6.0421	9.1046
	$-0.2{\bar{F}}_{\mathrm{cr}}^{E}$	3.7158	7.4427	7.3318	5.7344	8.8425
	$0.2{\bar{F}}_{\mathrm{cr}}^{E}$	3.3023	7.2449	7.0815	5.5188	8.6624
	$0.8{\bar{F}}_{\mathrm{cr}}^{E}$	2.5404	6.9360	6.6848	5.1770	8.3837

**Table 14:** Dimensionless fundamental frequency Ω for the AFG-GPLRC CS microbeam (${\bar{F}}_{\mathrm{cr}}^{E}=12.2065$).

AVLs	${\bar{F}}_{0}$	Epoxy	UD	AFG-X	AFG-O	AFG-V
*F_C_*	$-0.8{\bar{F}}_{\mathrm{cr}}^{E}$	18.6890	33.9065	31.5551	31.6508	33.7410
	$-0.2{\bar{F}}_{\mathrm{cr}}$	16.3024	32.6408	30.0920	30.3707	32.3107
	$0.2{\bar{F}}_{\mathrm{cr}}$	14.4760	31.7669	29.0723	29.4842	31.3144
	$0.8{\bar{F}}_{\mathrm{cr}}$	11.1458	30.4055	27.4662	28.0985	29.7461
*F_L_*	$-0.8{\bar{F}}_{\mathrm{cr}}^{E}$	19.3101	34.3092	31.9563	32.0265	34.4657
	$-0.2{\bar{F}}_{\mathrm{cr}}$	16.5106	32.7496	30.2006	30.4751	32.5097
	$0.2{\bar{F}}_{\mathrm{cr}}$	14.2120	31.6520	28.9573	29.3719	31.1015
	$0.8{\bar{F}}_{\mathrm{cr}}$	9.4039	29.9047	26.9615	27.5933	28.7957
*F_P_*	$-0.8{\bar{F}}_{\mathrm{cr}}^{E}$	19.7778	34.6448	32.2076	32.4312	35.0244
	$-0.2{\bar{F}}_{\mathrm{cr}}$	16.6835	32.8427	30.2712	30.5901	32.6702
	$0.2{\bar{F}}_{\mathrm{cr}}$	13.9726	31.5517	28.8806	29.2458	30.9239
	$0.8{\bar{F}}_{\mathrm{cr}}$	7.3098	29.4526	26.6104	27.0079	27.9525
*F_S_*	$-0.8{\bar{F}}_{\mathrm{cr}}^{E}$	18.0867	33.5910	31.4111	31.1437	33.2416
	$-0.2{\bar{F}}_{\mathrm{cr}}$	16.1384	32.5600	30.0565	30.2398	32.1809
	$0.2{\bar{F}}_{\mathrm{cr}}$	14.6534	31.8492	29.1075	29.6181	31.4480
	$0.8{\bar{F}}_{\mathrm{cr}}$	11.9970	30.7445	27.6048	28.6542	30.3061

**Figure 13:**
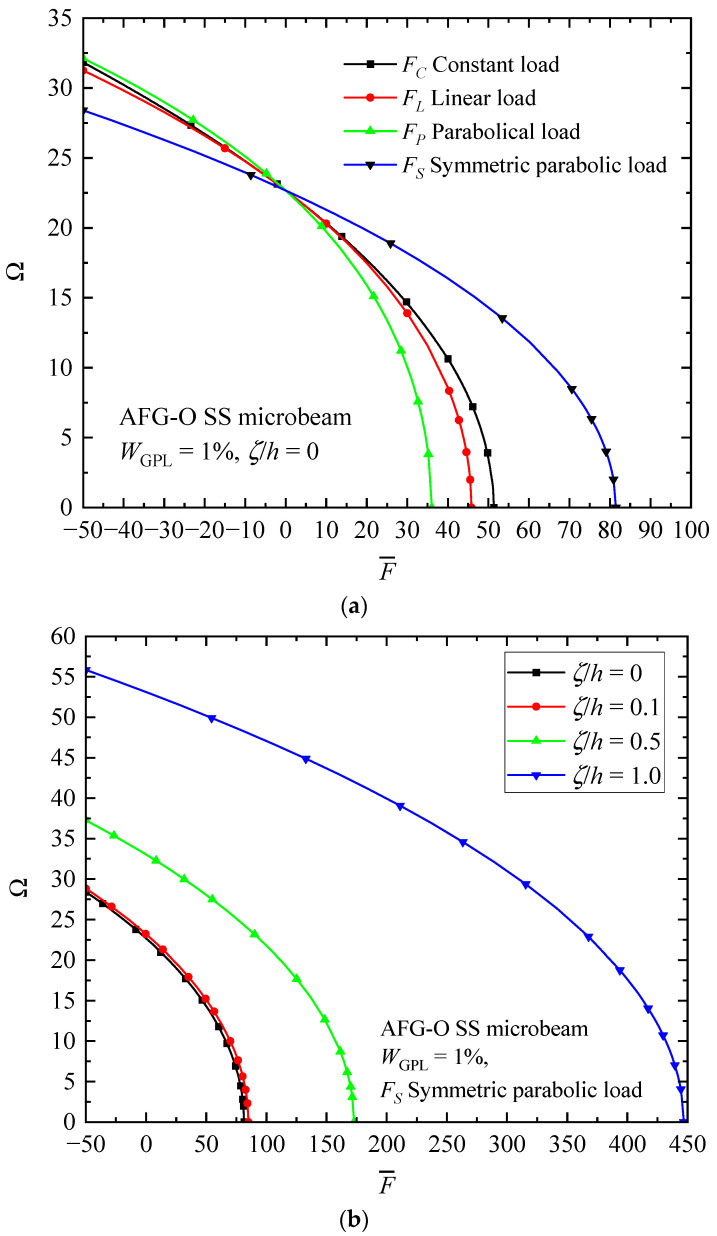
Effects of axially applied load on the fundamental frequency of AFG-O SS microbeams: (**a**) influence of axially variable load and (**b**) influence of MLSP to thickness ratio.

Figure 13 reveals the relationship between an axially applied load $\bar{F}$ and the fundamental frequency Ω of AFG-GPLRC microbeams. For the sake of brevity, only the simply supported AFG-O microbeam is analyzed under different types of axially variable loads and/or material length scale parameters. It is seen that the fundamental frequency decreases sharply with the promotion of the compressive load ($\bar{F}>0$). The fundamental frequency tends to approach zero when the axial load applied reaches the buckling load $\bar{F}={\bar{F}}_{\mathrm{cr}}$. In contrast, the frequency raises with tension loading ($\bar{F}<0$). Therefore, the compressive axial load decreases the beam stiffness, while the tensional load imposes an opposite influence. From the figure, it is again certified that the intrinsic size effect could dramatically promote the stiffness of the size-dependent microbeams, which, in turn, increases the vibration frequency and critical buckling load.

**Figure 14:**
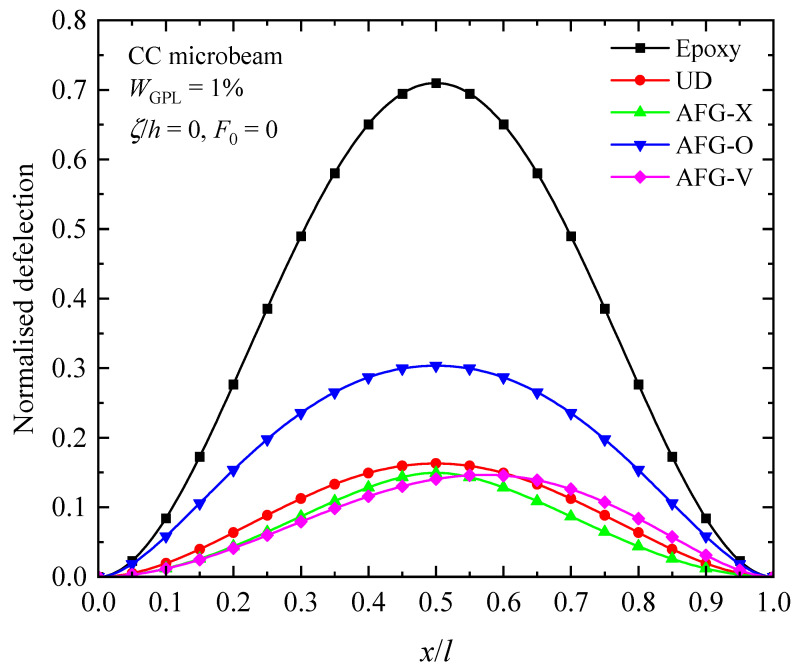
Fundamental vibration mode shapes of the AFG-GPLRC microbeam with respect to various axial GPL distribution patterns.

**Figure 15:**
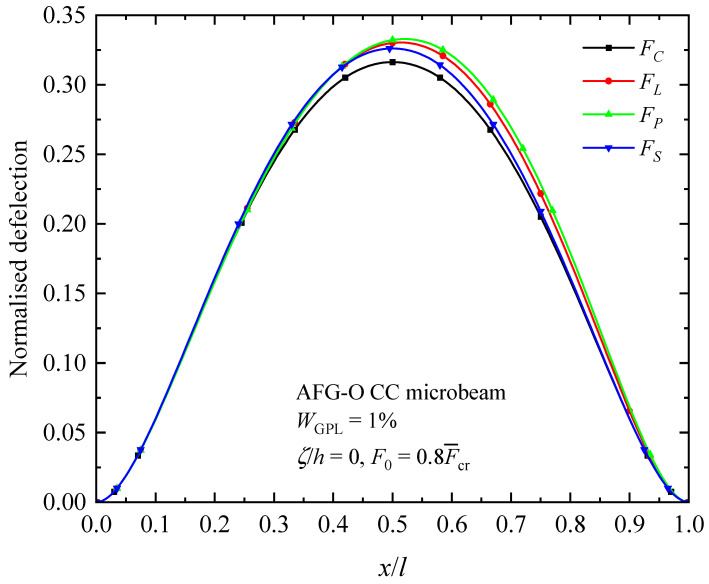
Fundamental vibration mode shapes of the AFG-GPLRC microbeam with respect to axially varying loads.

**Figure 16:**
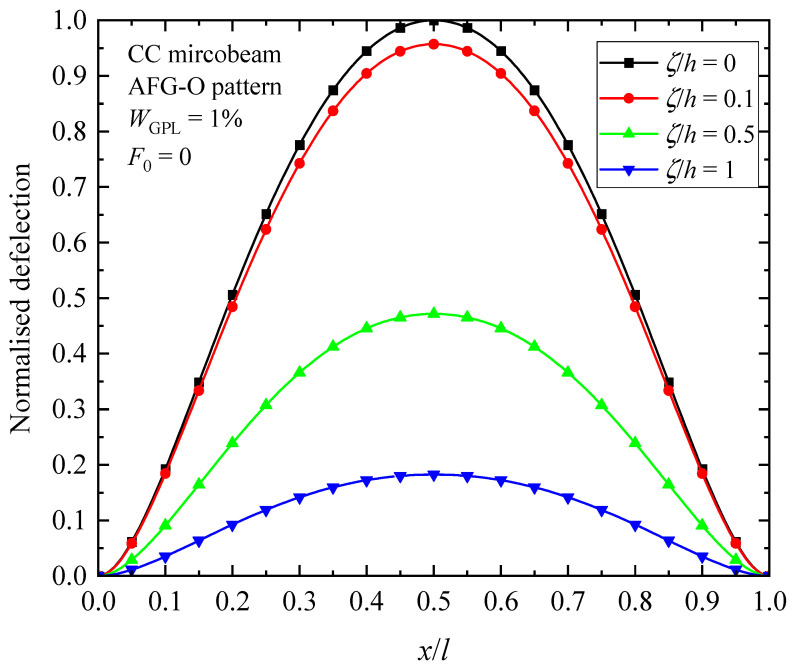
Fundamental vibration mode shapes of the AFG-GPLRC microbeam with respect to material length scale parameters.

Figure 14, Figure 15 and Figure 16 illustrate the changes in the shape of the fundamental vibration mode with respect to the axial GPL distribution, axially applied load, and material length scale parameters, respectively. Due to the comparability, only the CC microbeam is considered. It should be noticed that the axially graded distribution of GPL nanofillers plays a critical role in deciding the deflection of the AFG-GPLRC microbeams. The dispersion of GPLs increases the stiffness of the microbeam powerfully, and hence reduces the deformation. From Figure 12, it is seen that the deflection is symmetric except in the case of AFG-V, where the axial load is without consideration. However, Figure 13 shows that the vibration modes of microbeams with AVLs are asymmetric, even with a symmetric GPL distribution and boundary condition. This is because of the accumulation of axially applied loads like the buckling mode. Figure 14 demonstrates again that the size effect could promote the bending stiffness of the microbeams and decrease the peak of the vibration mode shape.

## 5. Conclusions

In the current work, a comprehensive theoretical analysis was developed to accurately predict the stability and free vibration performances of AFG-GPLRC microbeams under axially varying loads. The modified couple stress Euler–Bernoulli beam theory was utilized to derive the governing equation with the aid of the state-space method, and the discrete equilong segment model was adopted to solve the equation to evaluate the buckling loads and fundamental natural frequencies. The GPL nano-reinforcements dispersed into the polymer matrix material (epoxy) varied with weight fraction across the microbeam length, and the GPL-reinforced nanomaterial properties were calculated by employing the improved Halpin–Tsai micromechanics model and the rule of mixtures. Different types of AVLs were considered to check the buckling and vibration behaviors.

It is concluded:

1. The bending stiffness of the AFG-GPLRC microbeams can be powerfully promoted by the small-scale effect (the smaller, the stiffer). This small-scaled enhancement is due to the intrinsic size dependence of materials, and is more evident with a decrease in the size of the microbeams.
2. The addition of GPL nano-reinforcements shows promising results in improving the stability resistance and natural frequencies, and a few layers of single graphene sheets with larger surface areas can improve the beam stiffness more powerfully.
3. The axially graded effects of GPLs on the mechanical behaviors of AFG microbeams are decided by both the axial distribution pattern and boundary condition. For the CC and CS microbeams, the UD pattern achieves a much higher buckling resistance and fundamental frequency, while the AFG-V and AFG-O patterns are more suitable for the CF and SS microbeams, respectively.
4. The axial loading pattern also influences the buckling load and natural frequency of the microbeams significantly. The synergetic influence of AVLs on the buckling load and fundamental frequency, as well as modes, should be targeted in the design of microbeams.
5. Generally, the synchronized axial distributions of GPLs and applied load throughout the microbeam length could improve the buckling resistance and natural frequency more powerfully.

## Figures and Tables

**Table 1:** Load coefficients of AVLs.

Type of AVL	Symbol	Load Coefficients		
		$\alpha_{0}$	$\alpha_{1}$	$\alpha_{2}$
Constant load	*F_C_*	1	0	0
Linear load	*F_L_*	0	2	0
Parabolical load	*F_P_*	0	0	3
Symmetric parabolic load	*F_S_*	0	6	−6

**Table 2:** Material properties of the GPLs and epoxy [8].

Material Properties	GPLs	Epoxy
Young’s modulus (GPa)	1010	3.0
Mass density (kg m^−3^)	1060	1200
Poisson’s ratio	0.186	0.34

**Table 3:** Natural frequencies of AFGM beams with unit length.

Frequency Parameters	CC		CF		SS	
	Ref. [37]	Present	Ref. [37]	Present	Ref. [37]	Present
*μ*_1_	20.4721	20.4721	2.4256	2.4256	9.0286	9.0286
*μ*_2_	56.5482	56.5482	18.6041	18.6041	36.3715	36.3715
*μ*_3_	110.9396	110.9396	55.1791	55.1791	81.7289	81.7289
*μ*_4_	183.4447	183.4447	109.5748	109.5748	145.1907	145.1907

**Table 4:** The fundamental frequency parameter $\alpha^{4}=\rho A\omega^{2}l^{4}/EI$ for $\tau \left(0\right)=10$.

Parameters *γ*	CC		CF		SS	
	Ref. [36]	Present	Ref. [36]	Present	Ref. [36]	Present
100	5.8768	5.8768	3.5876	3.5876	5.0032	5.0032
4	5.0437	5.0437	2.7660	2.7660	3.8322	3.8322
−3	4.9587	4.9587	2.5956	2.5956	3.6689	3.6689

**Table 5:** The critical buckling parameter $\gamma_{c}$ for various $\tau \left(0\right)$.

BC	$\tau \left(0\right)=10$		$\tau \left(0\right)=4$		$\tau \left(0\right)=0$	
	Ref. [36]	Present	Ref. [36]	Present	Ref. [36]	Present
CC	92.3767	92.3767	81.7753	81.7753	74.6286	74.6286
CF	16.9986	16.9986	8.9816	8.9816	3.4766	3.4766
SS	35.5755	35.5755	25.5475	25.5475	18.5687	18.5687

**Table 6:** Dimensionless buckling load for the AFG-GPLRC SS microbeam (*W*_GPL_ = 1%).

AVLs	*ζ*/*h*	Epoxy	UD	AFG-X	AFG-O	AFG-V
Constant load *F_C_*	0	9.8696	43.0091	23.0554	51.3215	37.8012
	0.1	10.3115	44.9373	24.0883	53.6231	39.4956
	0.5	20.9177	91.2162	48.8766	108.8626	80.1601
	1.0	54.0619	235.8375	126.3402	281.4859	207.2369
Linear load *F_L_*	0	9.2792	40.4364	22.4870	45.8145	42.8317
	0.1	9.6947	42.2493	23.4944	47.8689	44.7522
	0.5	19.6664	85.7598	47.6722	97.1749	90.8432
	1.0	50.8280	221.7303	123.2279	251.2561	234.8778
Parabolical load *F_P_*	0	7.7171	33.6290	19.9703	36.0404	37.0528
	0.1	8.0626	35.1368	20.8650	37.6563	38.7143
	0.5	16.3557	71.3225	42.3381	76.4395	78.5899
	1.0	42.2713	184.4028	109.4418	197.6367	203.2014
Symmetric parabolic load *F_S_*	0	13.7960	60.1193	28.2999	81.4720	55.4054
	0.1	14.4138	62.8147	29.5675	85.1269	57.8890
	0.5	29.2393	127.5046	59.9912	172.8437	117.4960
	1.0	75.5692	329.6605	155.0654	446.9585	303.7676

**Table 7:** Dimensionless buckling load for the AFG-GPLRC CC microbeam (*W*_GPL_ = 1%).

AVLs	*ζ*/*h*	Epoxy	UD	AFG-X	AFG-O	AFG-V
Constant load *F_C_*	0	39.4784	172.0362	133.1470	137.4249	138.2338
	0.1	41.2461	179.7494	139.1161	143.5862	144.4303
	0.5	83.6707	364.8647	282.3745	291.4568	293.1452
	1.0	216.2475	943.3501	730.0568	753.5524	757.8794
Linear load *F_L_*	0	37.2950	162.5216	133.8917	125.1426	166.1325
	0.1	38.9650	169.8082	139.8946	130.7527	173.5825
	0.5	79.0432	344.6855	283.9660	265.3952	352.3804
	1.0	204.2877	891.1772	734.1890	686.1528	911.1240
Parabolical load *F_P_*	0	35.8365	156.1656	142.0906	109.5415	174.7855
	0.1	37.4411	163.1672	148.4619	114.4516	182.6248
	0.5	75.9519	331.2053	301.3735	232.2927	370.7690
	1.0	196.2983	856.3242	779.2222	600.5460	958.7194
Symmetric parabolic load *F_S_*	0	36.0861	157.2534	112.1374	147.2780	135.1531
	0.1	37.7019	164.3038	117.1642	153.8827	141.2120
	0.5	76.4810	333.5124	237.8083	312.3938	286.6266
	1.0	197.6657	862.2896	614.8210	807.7408	741.0472

**Table 8:** Dimensionless buckling load for the AFG-GPLRC CF microbeam (*W*_GPL_ = 1%).

AVLs	*ζ*/*h*	Epoxy	UD	AFG-X	AFG-O	AFG-V
Constant load *F_C_*	0	2.4674	10.7523	7.6922	7.9135	5.7632
	0.1	2.5779	11.2343	8.0369	8.2682	6.0213
	0.5	5.2294	22.8040	16.3097	16.7793	12.2177
	1.0	13.5155	58.9594	42.1623	43.3765	31.5813
Linear load *F_L_*	0	1.7357	7.5636	5.2802	6.0921	4.4571
	0.1	1.8134	7.9028	5.5168	6.3651	4.6568
	0.5	3.6786	16.0414	11.1955	12.9181	9.4494
	1.0	9.5074	41.4747	28.9414	33.3963	24.4262
Parabolical load *F_P_*	0	1.4886	6.4868	4.4517	5.4225	4.0354
	0.1	1.5552	6.7776	4.6512	5.6655	4.2162
	0.5	3.1549	13.7576	9.4387	11.4986	8.5556
	1.0	8.1538	35.5700	24.3998	29.7269	22.1163
Symmetric parabolic load *F_S_*	0	2.4416	10.6396	8.1135	7.5923	5.4371
	0.1	2.5509	11.1166	8.4771	7.9325	5.6806
	0.5	5.1746	22.5651	17.2037	16.0979	11.5261
	1.0	13.3739	58.3417	44.4741	41.6148	29.7934

**Table 9:** Dimensionless buckling load for the AFG-GPLRC CS microbeam (*W*_GPL_ = 1%).

AVLs	*ζ*/*h*	Epoxy	UD	AFG-X	AFG-O	AFG-V
Constant load *F_C_*	0	20.1907	87.9857	69.2723	80.5295	72.1372
	0.1	21.0948	91.9305	72.3777	84.1401	75.3708
	0.5	42.7923	186.6053	146.9061	170.7942	152.9766
	1.0	110.5970	482.4642	379.8072	441.5885	395.4948
Linear load *F_L_*	0	14.9841	65.2967	54.7540	57.8374	62.8773
	0.1	15.6550	68.2243	57.2086	60.4303	65.6965
	0.5	31.7574	138.4851	116.1183	122.6585	133.3583
	1.0	82.0772	358.0504	300.2110	317.1216	344.8012
Parabolical load *F_P_*	0	12.2065	53.1928	47.3576	44.9621	53.5784
	0.1	12.7531	55.5777	49.4807	46.9775	55.9809
	0.5	25.8706	112.8145	100.4359	95.3490	113.6412
	1.0	66.8627	291.6795	259.6709	246.5096	293.8294
Symmetric parabolic load *F_S_*	0	23.7027	103.2901	70.3261	109.9998	84.1200
	0.1	24.7640	107.9210	73.4782	114.9332	87.8907
	0.5	50.2356	219.0637	149.1294	233.3343	178.3864
	1.0	129.8344	566.3846	385.5393	603.3380	461.1856

## Data Availability

Data are contained within the article.
